# Conference Report: QUEST FOR EXCELLENCE VIII Washington, DC February 5–7, 1996

**DOI:** 10.6028/jres.101.043

**Published:** 1996

**Authors:** Cap Frank

**Affiliations:** Office of Quality Programs, National Institute of Standards and Technology, Gaithersburg, MD 20899-0001

## 1. Introduction

Nine years ago, the American business community and government formed a partnership to promote a national quality improvement campaign and established the structure and endowment launching the Malcolm Baldrige National Quality Award. The Award, created in 1987 by public law, continues to promote an understanding of quality business practices, greater awareness of continuous improvement as a crucial competitive element, the sharing of information and successful strategies through publications, such as the Winners’ Profiles, and public activities. The most prominent of these activities is the annual Quest for Excellence Conference.

Each year, the Quest for Excellence Conference, the official conference of the Malcolm Baldrige National Quality Award, provides a forum for quality-conscious business leaders worldwide to hear and question the most recent Baldrige Award recipients. For the last 8 years, business executives have come to this conference to learn about the quality journeys of Award-winning companies and how to adapt and implement total quality management practices to their own organizations. Conference participants view the current and prior Award winners as quality practices benchmarks for American business.

The eighth annual Quest for Excellence Conference was held in Washington, DC (February 5–7, 1996), operated under the joint sponsorship of the American Society for Quality Control (ASQC), the Association for Quality and Participation, the Council on Competitiveness, and the National Institute of Standards and Technology’s Office of Quality Programs. The conference provided a number of firsts for the audience of 1100. This was the first time the current Baldrige winning companies were honored in a conference kick-off ceremony at which each company received the Baldrige flag. It was the first Quest for Excellence not led by the first Director of the Baldrige Program, Dr. Curt W. Reimann,^1^ who retired on December 15, 1995. For the first time, Baldrige recipients traced their roots to the middle of the last century. Another first was that both Award winners mentioned that they embraced the Baldrige Criteria from positions of strength rather than from some perceived or explicit organizational weakness.

This year’s conference featured the two 1995 winners of the Malcolm Baldrige National Quality Award: Armstrong World Industries, Inc., Building Products Operations (BPO), and the Telecommunications Products Division of Corning Incorporated (Corning TPD). Both were winners in the manufacturing category.^2^ During the 3-day conference, attended by domestic and foreign representatives^3^ from business, education, health care, and government, personnel from each of the Award recipients addressed full conference plenary sessions and smaller group discussion and question-and-answer breakouts.^4^ All sessions provided significant time for audience interaction. Conference attendees were encouraged to query Baldrige winning companies during the breakouts to learn details of their approaches to addressing each of the seven Baldrige Award Criteria Categories.^5^

BPO is the world’s largest manufacturer and marketer of acoustical ceiling systems for commercial and residential markets. In 1994, sales totaled $628 million. BPO U.S. operations has 2500 employees working at its headquarters in Lancaster, PA, and in seven manufacturing facilities: Beaver Falls and Marietta, PA; Hilliard, OH; Mobile, AL; Macon, GA; Pensacola, FL; and St. Helens, OR. Each year for the past 5 years, BPO has had more than 250 improvement teams operating at any given time. “Best practices” generated by these teams are shared among plants through conference calls, computer networks, and “Functional Excellence” conferences. Output per manufacturing employee and annual sales per manufacturing employee are critical measures of performance for BPO. As a result of employee involvement, recognition, gainsharing, and eliminating “non-value-added” activities, since 1991 output per manufacturing employee has improved by 39 % and annual sales per manufacturing employee have risen by 40 %.

Using technology developed and patented by its parent, Corning Incorporated, Corning TPD was formed in 1983 to manufacture optical fiber that uses pulses of light to carry large amounts of information over great distances at a low cost.^6^ With sales in 30 different countries, Corning TPD is the largest optical fiber manufacturer in the world. The company has formed joint ventures with companies in Europe and Australia to manufacture optical fiber. Secretary of Commerce Ronald H. Brown^7^ observed that Corning TPD’s success with foreign joint venturing took “full advantage of the export market in ways that all of our companies can learn from.” Nearly 1200 of Corning TPD’s 1400 employees work in its single manufacturing facility in Wilmington, NC. The remainder are in its headquarters’ operation in Corning, NY.

Corning TPD uses a formal, documented process management system to control and continuously improve over 800 processes throughout its business and manufacturing operations. Fifty of these processes are designated “Core Business Processes” and are “owned” and managed by Corning TPD’s key business leaders. One measure of satisfaction is the quality of products shipped to customers, expressed as parts-per-million product returns. In 1994, returns were at or below the 250 parts-per-million level, meaning that only 250 reels of fiber out of every 1 million reels were returned.

In addition to the 1995 Winners’ presentations on “Lessons Learned” and “Category 7: Customer Focus and Satisfaction,” this year’s conference devoted the final day to two additional concurrent sessions. These additional sessions were an all day meeting for each of the two 1995 pilot programs, health care and education, and an afternoon session on state and local quality awards.

## 2. Leading the Quality Journey and Lessons Learned

Dr. Harry Hertz^8^ opened the conference welcoming the two newest members to the family of Baldrige Award recipients and introduced the keynote speaker, Secretary Brown, as one of the key champions of American competitiveness. After recognizing the 1995 Malcolm Baldrige National Quality Award winners, BPO and Corning TPD, Secretary Brown opened his talk with, “I firmly believe, and I am sure that you share this belief, that America’s success in the 21st century will depend on our commitment as a nation to the principle of excellence. As we look around the room, we can see America’s advantages in the battle for global competitiveness … our open and diverse culture, our undisputed leadership in many areas of the key industries of the future, our talented, adaptable work force, our innovative, dynamic firms, our desire to work in partnership to get done what none of us could do alone. Already these advantages are enabling us to command new economic opportunities and regain our competitive edge in international markets. For the second straight year, in fact, America tops the list of the world’s most competitive economies ahead of both Japan and Germany, according to the Geneva-based World Economic Forum.” The Secretary went on to note that, due to this country’s competitive edge, many foreign countries, such as Singapore, Mexico, and Sweden,^9^ are basing their national awards on the Baldrige Award Criteria.

“The 1995 Baldrige Award Winners,” Secretary Brown observed, “exemplify just how important it is to stretch for lofty goals. They also illuminate the rest of us as we find pathways to success in the 21st century … investments in people, in innovations, and in public-private partnerships.” Secretary Brown illustrated his point by highlighting BPO’s innovations—its computer-based, three-dimensional product modeling system, maximum use of recycled materials, safety record, and partnering arrangements. He also complimented Corning TPD on its four-pronged “Plan to Win,” 98 % customer satisfaction track record, dramatic reduction of defects, and significantly increased productivity.

Citing the strong track record of business community success with the Baldrige Award, Secretary Brown regretted the lack of Congressional support for the health care and education pilot programs which adapted the Baldrige Criteria for use by American organizations from these two important sectors.

Honoring the retirement of Dr. Curt W. Reimann, Secretary Brown characterized Dr. Reimann as a hard working, dedicated, and underappreciated civil servant. The Secretary’s comments provoked a standing ovation by the audience.

Noting that the annual public investment of less than $3 million dollars in the Baldrige Program has leveraged over $100 million dollars in private sector benefits, Secretary Brown observed the rarity of this achievement as private and public investment in research and development (R&D) have been anemic with alarming downward trends during this last decade. Secretary Brown stated that this decline in American R&D and the declining support on the part of the U.S. Congress of public-private R&D partnerships “ignores the realities of the marketplace where public and private investments in technology and innovation since World War II have been responsible for as much as one half of our nation’s economic growth. This is at a time when countries such as Japan are increasing their public investment in and encouragement of industrial research and development. Using Japan as just one example of a positive government climate, each year the country invests 35 % more than the United States on a per capita basis and recently announced plans to double the country’s R&D efforts investment by the year 2000.”

Secretary Brown punctuated his keynote address by asking the leaders of this year’s Baldrige winning companies to come up on stage to receive the official Baldrige flag. Secretary Brown presented a Baldrige flag to BPO’s representatives, Henry A. Bradshaw, President, Worldwide Building Products Operations, and John F. McClay, Manager of Quality Management. From Corning TPD, Robert C. Forrest, Senior Vice President and General Manager, and Gerald J. McQuaid, Division Vice President and Director Business Systems, accepted the Baldrige flag. These flags are flown with pride over the headquarters and operations of Baldrige Award recipients. Another standing ovation led the recipients off the stage and ushered in the premier showing of the latest Quest for Excellence videotape.^10^

At the close of the videotape, Dr. Hertz introduced the leadership section of the conference by noting that, “Leadership is the focal point within the Criteria for a company to describe its values, performance expectations, and leadership system to create and sustain customer focus and to achieve expectations. The personal involvement of senior leadership is critical in developing and maintaining the leadership system. Leadership sets the tone for the company’s contribution as a corporate citizen and for meeting its public responsibilities.”

The conference’s first speaker, BPO’s Henry Bradshaw, thanked the Baldrige examination team, “who spent the better part of last year learning about us and evaluating our strengths and areas for improvement. During our site visit, the examiners were tenacious in their quest for facts, but they were also considerate guests in our house.”

“Armstrong’s leaders—including me—have attended Quest conferences since they began with the 1988 Baldrige Award winners, and we’ve learned from every one of them. One very important lesson we’ve learned is that to advance your quality culture, you need a leader who does two things: first, understand your quality culture so you can continue to change it for the better. Second, know how to build on your organization’s existing strengths to develop new capabilities.

“Without those two elements of leadership,^11^ you simply cannot sustain and build your quality culture. The values our leaders have promoted since the 1860’s and first written on paper during the company’s 100th anniversary are: We respect people, we maintain high moral and ethical standards for ourselves, we show good taste and courtesy, and we serve our stakeholders fairly and in proper balance.”

Robert Forrest described leadership at Corning TPD as going beyond the traditional notion of leadership setting and communicating direction. At Corning TPD, leadership also provides the creation of linkages (such as between TPD people and TPD business objectives) and ensures integration of the “Plan to Win”^12^ (a six-element prescription for actions). Leadership is central to Corning TPD’s quality journey.

Gerald J. McQuaid described Corning TPD’s quality journey as three parallel and interconnected tracks: motivation, systems, and infrastructure. He noted, “In a simpler and more tidy world, you would sort out your motivation, select your system’s approach, and go about building the required infrastructure sequentially. It didn’t happen that way for us, and it may not be happening that way for you.” McQuaid noted that these parallel tracks evolved through four distinct phases: product focus, total quality, cherry picking, and integration.

McQuaid observed that early in the quality journey, TPD had “one foot in the Baldrige boat and one foot in the business as usual boat.” With no real wake-up call, McQuaid explained that TPD “cherry picked” its way through its first Baldrige feedback report (1989), picking and choosing which comments to work on and which to table. By 1993, it became clear that TPD could not continue with feet in different boats. Business was good and complacency was becoming a strong possibility. This began TPD’s integration phase, a nonlinear weaving of the Baldrige Criteria into the fabric of everyday TPD life, and McQuaid noted that is where Corning TPD is still today.

Robert Forrest supplemented McQuaid’s comments with, “Our decision to adopt the Baldrige Criteria in 1989 was not motivated by a wake-up call, we were not in trouble. The decision was business driven through and through. It [the Baldrige Criteria] was the best business model we had found. The feedback from our first Baldrige application in 1989 provided the basis for many of the systems in place today. The 40-page feedback report we just got from our winning application will form the foundation for future improvements.”

Quality needs to be integrated into the basic business fabric. As Forrest explained, “Let me warn you, you won’t hear our people mention the word “quality” very often. If you ask them to show you Corning TPD’s quality strategy, they will share with you our business strategy. If you want to look at our quality plans, they will take you through our business plans. If you ask them what their quality objectives are, they will show you their business objectives. And if you ask me for our quality organization chart, I’ll give you the name of everyone in TPD. Business and quality, at TPD, they’re not two separate things. They’re neither complementary nor supplementary. Rather they are one and the same, integrated and indistinguishable. In our experience, that’s how it works best and that’s how we won the Baldrige Award. And that’s how we win every day in the optical fiber industry.

“People ask us how do we have the time or make the time for quality. My answer is if you think about quality stuff, then you are in trouble. You have to think about business stuff. Quality has to be integrated into the business fabric and all business factors, otherwise quality will not take hold.”

Describing lessons learned on the way to winning the Baldrige Award, L. R. Kishpaugh, Corning TPD’s Manager of Customer Services, observed: “When your quality objectives become your business objectives, when your quality teams become your business teams, when your quality award programs are replaced with business-linked reward systems, then you’ll know you’re integrated.”

Kishpaugh also cited increased customer focus as another lesson learned. “.… our customers seemed satisfied, and we were making money. As the saying goes: if it ain’t broke, why fix it? Thanks to Baldrige, however, we discovered places where it wasn’t broken, but had developed some pretty serious cracks. Customer focus was one of the areas with developing cracks. Our response led to increasing our customer focus, not just in sales and customer service, but also in new product development and a number of business processes.”

Another lesson Corning TPD learned was that people need to feel valued and that it was easy to lose sight of people in a highly technological business. Kishpaugh concluded, “No matter how technical your product, no matter how advanced your process, every business begins with a valued person.”

John McClay, Armstrong BPO’s Manager of Quality Management, noted this as being his third Quest For Excellence conference. At the first one, in 1989, after listening to Globe, Westinghouse, and Motorola,^13^ McClay remembers, “I was amazed by what they’d accomplished. I felt as if those people were playing in the major leagues of quality, and my team was working on the basics, the kinds of things you learn in little league. The second time I attended the Quest, four years later in 1993, there were five winners of the 1992 Baldrige Award, and I remember thinking that we’ve come a long way. I felt that we were also playing in the major leagues.”

“I see the Quest for Excellence conferences as a great way to informally assess your company’s progress against the Baldrige Criteria. When you hear what Baldrige-winning companies are doing, and where they’ve been on their quality journey, you get a “big picture” view of where you are and how far along your company has come.” McClay described the five phases that BPO took in its quality journey from instituting a Philip Crosby style quality management system in 1983 to the present Phase V, Business Excellence Process, and its three components to drive, process, and support changes.

William B. McBee, BPO’s Director, Corporate Quality, spoke about lessons learned at BPO and observed that the first hurdle was to decide how to measure the overall effectiveness of the quality interventions. According to McBee, the Baldrige Criteria were given a trial test to learn if they could “improve our ability to manage and even accelerate the speed of our improvement process.” This was a hard won trial test since Armstrong’s Corporate Quality Council (with business leaders and corporate staff heads forming the membership) had concerns such as: the Baldrige Award Criteria were seen as a foreign language, and the council noted that “we don’t need more jargon to add noise in the channel.” The Criteria represented additional work, and “we were already lean and working long hours.” The value of the Criteria was not apparent up front, “like jogging, eating right, lifting weights, benefits are down the road.” The fact that the Criteria were U.S.-based made selling their use to “our European operations very difficult.”

In 1989, the Corporate Quality Council reached an agreement to experiment with the Criteria as a self-assessment tool in 30 operations. “But,” according to McBee, “I can tell you it was Baldrige on trial.” The Baldrige-based self-assessments revealed both opportunities for improvement and a clearer definition of patterns of strengths and weaknesses. Armstrong learned more about leadership, quality values, customer input, and more challenging goals. McBee noted that observing the senior leadership of previous Baldrige winners “helped us develop our own stretch goal program we called 80 in 5.” Along with stretch goals went benchmarking, and McBee noted that “benchmarking was a relatively new term for us in 1990 as we were not systematically seeking best practices and benchmarking data to set meaningful goals.”

McBee concluded that “the types of changes we made as a result of our Baldrige Criteria assisted self-assessment and had a direct impact on both our business and the speed of our culture change.” “Another important lesson learned,” according to McBee was that, “the value of using the Criteria is proportional to how willing and able leadership is to act upon the feedback. We were fortunate. Our leaders were not defensive and took a personal role in leading the changes.”

According to McBee, BPO’s first Baldrige application in 1993, following the experience of a 1992 application to Armstrong’s internal award process, was written by those closest to the work, and afforded BPO a site visit, which provided several important lessons. “Our human resource strategy was not fully deployed, we needed to make better use of competitive comparisons and outside benchmarks to provide a rationale for our stretch goals, and our corporate staff groups were not as closely focused as they should be on BPO’s quality and performance improvement plans. Addressing these weaknesses^14^ were both strategic and tactical and we made the decision not to apply in 1994 to give us time to move forward. But the criteria continued to be catalysts for change. As a result of this early experience and applying for the Baldrige in 1995, our stretch goals became more fact-based and more rational targets, and we changed our focus from customer satisfaction to customer value. We found that the pursuit of ever higher levels of customer satisfaction did not always translate into improved market share or business results…” McBee reminded the audience that the bottom-line was the proof of shareholder value and that Armstrong stock appreciated 94 % in the last 3 years (while the Standard & Poors returned 41 %).

Looking around the room as he closed his address to the full conference audience, McBee shared, “Looking back at how we navigated our way through the quality journey, I have one last lesson learned. One does not become world-class by just focusing on weaknesses and correcting them. If you’re not careful, you may still end up an average company with less problems. To leverage your improvement efforts, focus on your strengths and take those to new heights. The Baldrige Criteria tell you the difference.”

When Henry Bradshaw was asked what’s next after winning the Malcolm Baldrige National Quality Award, his answer provided an effective wrap-up to this year’s Quest for Excellence Conference and a conclusion to this report. Henry Bradshaw responded, “…for the same reasons that we started in 1983 with the quality management process. We knew companies were getting better at that time and, although we were a leader in our industry, we wanted to maintain that leadership. I read somewhere that those who won the Baldrige in the past have only scored around 700 points. That means that there are 300 more points yet that we can work on, and we are definitely going to do that by continuously improving all our processes.”

## Footnotes

1 At the close of 1995, Curt Reimann stepped down as Director of the Malcolm Baldrige National Quality Award Program and retired after 33 years of federal government service. During Dr. Reimann’s 8 year stewardship as director, the Malcolm Baldrige National Quality Award has become the centerpiece of the nation’s quality and competitiveness initiative. Conference organizers hosted a pre-conference reception honoring Dr. Reimann’s singular contributions to the Malcolm Baldrige National Quality Award as its first Director and principal author of all previous versions of the Award Criteria. Highlights of the retirement reception, attended by Commerce Secretary Ronald H. Brown, were the gifts presented by Letitia Baldrige on behalf of the Baldrige Family (an elaborately tooled cowboy belt buckle won by the late Malcolm Baldrige at a rodeo), Baldrige winning companies (an endowed quality management scholarship at the University of Michigan), and the Foundation for the Malcolm Baldrige National Quality Award (a Tiffany crystal vase). (Refer to Appendix A for more information about the career of Curt Reimann.)

2 There are three categories in which American companies may compete: manufacturing, service, and small business.

3 In attendance were representatives from Brazil, Canada, Chile, England, Finland, Japan, Korea, Peru, and Sweden.

4 Audio tapes of all plenary, workshops, and most breakout sessions are available.

5 The Baldrige Award Criteria (continuously evolving through each of the 9 years of the program’s existence) provide a framework against which companies assess their continuous improvement efforts and management systems. (Refer to Appendix B for additional Criteria information.)

6 Optical fiber is replacing copper wire as the preferred method of transmitting information. Optical fiber can transmit error-free data, voice, image, fax, and video all through the same network. Two strands of this hair-thin glass have the same information-carrying capacity as a copper cable 10 cm thick. A single fiber, a thickness of one human hair, can transmit the entire contents of the 44 million word *Encyclopedia Britannica* and 770 000 words of the King James and New Testament Bibles around the world in just 2 seconds.

7 Secretary Brown was killed in an airplane crash in Croatia on April 3, 1996, while leading a delegation of government and senior leaders on a mission to aid in the rebuilding of the war-torn Balkans region.

8 Dr. Hertz is the current Director of the National Institute of Standards and Technology’s Office of Quality Programs, the federal program which manages the Malcolm Baldrige National Quality Award. (At the time of the conference, Dr. Hertz was the Acting Director and the Baldrige Program’s long-time Deputy Director.)

9 Even Japan is creating a Baldrige Criteria-based national award to complement the Deming Prize.

10 Each year, a videotape is produced which profiles the current Baldrige winning companies and highlights their results and best business practices.

11 This leadership system evolved from its 1983 roots at around the same time that two of the 1994 Baldrige Recipients, AT&T and Wainwright Industries, started their team building and quality improvement activities.

12 Robert Forrest described “Plan to Win” as an approach to “integrate our business, and it sits at the center of our world, energizing our day-to-day activities.” The six elements of “Plan to Win” are: strategy driven behavior, customer focus, managing by processes, continuous improvement, measure with the Baldrige Criteria, and build on the foundation of the Corning’s TPD’s three values of people, process, and technology (“…the constants that bind us together in an ever changing world,” according to Forrest).

13 Globe Metallurgical, Inc. (manufacturing), Westinghouse Electric Corporation Commercial Nuclear Fuel Division (manufacturing), and Motorola, Inc. (manufacturing) were the inaugural (1988) Malcolm Baldrige National Quality Award Winners.

14 The weaknesses which Mr. McBee mentions here are areas for improvement cited in a feedback report from the Baldrige Award Program summarizing the findings of a team of Baldrige examiners assessing BPO’s application and a fact-finding site visit.

## 3. Appendix A. Curt W. Reimann

Dr. Curt W. Reimann created a focal point for business leaders and government officials to realize a true national partnership. Before his efforts to bring groups together, quality and competitiveness initiatives were fragmented. The thoroughness of approach and rigor of the process that Dr. Reimann developed and insisted on were critical to the establishment and worldwide acceptance of America’s National Quality Award.

With the Award’s credibility established, Award winners and many dedicated volunteers joined the effort initiated by an Act of Congress and brought to life by Dr. Reimann. The Malcolm Baldrige National Quality Award Program has distributed over one million copies of the Award Criteria; over 10 000 presentations have been made by Award winners; and the Baldrige concept has been expanded to education, health care, and government sectors, other nations around the globe, and over 50 state and local award programs.

Dr. Reimann has significantly advanced the understanding, trust, and cooperation between business and government at a time when competitiveness has come to the attention and forefront of the national economic well-being. The 1996 Award Criteria were Dr. Reimann’s last major official Baldrige Program responsibility.

Prior to becoming Director of the Malcolm Baldrige National Quality Award Program, Dr. Reimann, a Queens, NY, native and University of Michigan alumnus, served as the Deputy Director of the National Institute of Standards and Technology National Measurement Laboratory, the Director of NIST’s Center for Analytical Chemistry, and a Research Chemist on the staff of the National Bureau of Standards (NIST’s predecessor organization).

For his contributions, Dr. Reimann has received the United States Department of Commerce Gold Medal, the American Society for Quality Control Award of Merit, and the government’s Presidential Distinguished Rank Award. He was also recognized by *Quality Review* as one of the “ten most influential figures” in quality, and in 1993, he was named a Fellow of the World Academy of Productivity Science.

Upon Dr. Reimann’s retirement, the Malcolm Baldrige National Quality Award Program has been placed in the capable hands of Dr. Harry S. Hertz, the program’s Deputy Director. Dr. Hertz, a 22-year veteran of federal service, also hails from the NIST chemistry program and senior NIST leadership positions.

## 4. Appendix B. The Award Criteria

Business leaders note that in addition to being the basis for Award selection and for giving feedback to Award applicants, the Malcolm Baldrige National Quality Award Criteria hold a significant leadership role in strengthening U.S. competitiveness by:

- helping to improve performance practices and capabilities;
- facilitating communication and sharing of best practices information among and within organizations of all types based upon a common understanding of key performance requirements; and
- serving as a working tool for managing performance, planning, training, and assessment.

The Criteria have as their foundation a set of core values and concepts. Excerpting from the 1996 Malcolm Baldrige National Quality Award Criteria publication, the core values are: customer-driven quality, effective senior leadership, continuous improvement and learning, employee participation and development, fast response and ever-shorter cycle times, design quality and waste prevention, long-range view of the future, management by fact, partnership development, corporate responsibility and citizenship, and results orientation. These core values and concepts are embodied in the seven Categories of the Award Criteria: Leadership, Information and Analysis, Strategic Planning, Human Resource Development and Management, Process Management, Business Results, and Customer Focus and Satisfaction.

As a component of its outreach and education mission and to ensure that the Malcolm Baldrige National Quality Award continues to be a catalyst for change, the National Institute of Standards and Technology’s Office of Quality Programs, which manages the Award, is available to assist states and localities to adopt Baldrige-based criteria and application assessment procedures for their own recognition programs. As of the date of the conference, 51 state, Senate Productivity, and local quality award programs were in operation in 38 states. Most are modeled after the Baldrige Award. To further broaden the impact of the sharing of best management practices, the Baldrige Program had 1995 pilot efforts expanding quality criteria to the education and health care communities.

## 5. General References

1995 Criteria for the Malcolm Baldrige National Quality Award.
Conference notebook, Quest for Excellence VIII, February 5–7, 1996, Washington, DC.
Audio Archives International tapes of the conference.

